# A new approach to assess gambling-like behavior in laboratory rats: using intracranial self-stimulation as a positive reinforcer

**DOI:** 10.3389/fnbeh.2014.00215

**Published:** 2014-06-11

**Authors:** Stephanie E. Tedford, Nathan A. Holtz, Amanda L. Persons, T. Celeste Napier

**Affiliations:** ^1^Department of Pharmacology, Center for Compulsive Behavior and Addiction, Rush University Medical CenterChicago, IL, USA; ^2^Department of Pharmacology, Rush University Medical CenterChicago, IL, USA; ^3^Department of Psychiatry, Rush University Medical CenterChicago, IL, USA

**Keywords:** cost/benefit decision-making, discounting, effort-based decision-making, gambling, intracranial self-stimulation

## Abstract

Pathological gambling is one manifestation of impulse control disorders. The biological underpinnings of these disorders remain elusive and treatment is far from ideal. Animal models of impulse control disorders are a critical research tool for understanding this condition and for medication development. Modeling such complex behaviors is daunting, but by its deconstruction, scientists have recapitulated in animals critical aspects of gambling. One aspect of gambling is cost/benefit decision-making wherein one weighs the anticipated costs and expected benefits of a course of action. Risk/reward, delay-based and effort-based decision-making all represent cost/benefit choices. These features are studied in humans and have been translated to animal protocols to measure decision-making processes. Traditionally, the positive reinforcer used in animal studies is food. Here, we describe how intracranial self-stimulation can be used for cost/benefit decision-making tasks and overview our recent studies showing how pharmacological therapies alter these behaviors in laboratory rats. We propose that these models may have value in screening new compounds for the ability to promote and prevent aspects of gambling behavior.

## Introduction

Problem or maladaptive gambling, including the extreme condition termed pathological gambling, is characterized by behaviors that often persist over extended periods. Problem gambling can have a significant negative impact on personal, professional and financial well-being. In the last two decades, gambling opportunities have increased through changes in legislation and the introduction of new venues (e.g., internet gambling). Accordingly, the prevalence of problem gambling has been on the rise. There are no FDA-approved treatments for this disorder, and thus, it is critical to better understand these behaviors in order to develop efficacious therapies.

Problem gambling is a complex phenomenon, which includes increased levels of impulsive decision-making (Alessi and Petry, 2003; Dixon et al., 2003; Holt et al., 2003; Kraplin et al., 2014) that stem from disadvantageous evaluations of cost/benefits. Clinical assessments of decision-making, which often employ survey and interactive computer-based tools, have been instrumental in determining suboptimal decision-making profiles in various pathologies including pathological gamblers (Ledgerwood et al., 2009; Madden et al., 2009; Michalczuk et al., 2011; Petry, 2011; Miedl et al., 2012). Clinical assessments are frequently made based on three differing, albeit overlapping, aspects of cost/benefit decision-making, including the following: (i) the amount of risk in obtaining a reward (risk/reward decision-making), (ii) a delay experienced before reward delivery (delay-based decision-making), and (iii) the amount of effort required to obtain a reward (effort-based decision-making). Several tasks have been developed to measure these critical features of suboptimal decision-making to further understand processes that comprise problem gambling. In these tasks, the subject chooses between a small and large reward, each associated with specific response contingencies. In risk/reward decision-making (i.e., probability discounting), subjects choose between a small reward delivered consistently at high probabilities (e.g., 100% probability of receiving $10) and a large reward delivered at varying probabilities (e.g., 10–80% probability of receiving $100). In clinical and preclinical studies, the absence of an expected reward is an aversive event which elicits corresponding physiological responses (Douglas and Parry, 1994; Papini and Dudley, 1997). Preference for the larger, “risky” option over the small, certain option is considered to reflect suboptimal risk/reward decision-making, and has been reported for several human pathologies that display enhanced impulsivity (Reynolds et al., 2004; Rasmussen et al., 2010; Dai et al., 2013). In delay-based decision-making (i.e., delay discounting, a measure of impulsive choice), the small reward is delivered soon after the option is selected, whereas the large reward is delivered following a variable delay, (e.g., $10 now or $100 in 2 weeks). Individuals who exhibit high impulsivity demonstrate preference for immediately available rewards (even if smaller), over delayed rewards (even if larger) although the latter option may be more beneficial to the individual (Crean et al., 2000; Reynolds et al., 2004; Bickel et al., 2012). In effort-based decision-making, the subject chooses between a small reward delivered following small amounts of effort, or a large reward delivered after a greater amount of effort has been exerted. In this task, individual preference for the high effort/large reward option and the “point” at which the individual switches to the low effort/small reward option is determined. Studies of effort-based decision-making in human gamblers have yet to be conducted, but would be of significant interest to assess cognitive function in this population.

Decision-making protocols used in clinical assessments can be modified to study decision-making in laboratory rats, and these models are critical for exploring the behavioral and neuropharmacological aspects of pathological gambling. In rats, decision-making can be assessed by placing the animal in an operant conditioning chamber, and allowing the animal to choose between two levers (or two nose-poke hoppers) that are made available at the same time. The established reward modality for the positive reinforcer in these rodent tasks is food (Stopper and Floresco, 2011; Eubig et al., 2014). We discuss here a novel method used in our laboratory which employs direct electrical stimulation of brain reward pathways (intracranial self-stimulation; ICSS) to assess cost/benefit decision-making in rats and the contribution of monoaminergic neurotransmitters in decision-making (Rokosik and Napier, 2011, 2012; Tedford et al., 2012; Persons et al., 2013).

## Intracranial self-stimulation

An operant reinforcer is a stimulus, which when made dependent upon some action, increases the likelihood of the recurrence of that action. Intracranial self-stimulation (ICSS) is an operant behavior in which animals self-administer electrical stimulation to brain regions known to be involved in positive reinforcement. ICSS was first studied in the 1950s when James Olds and Peter Milner (Olds and Milner, 1954) determined that rats would repeatedly return to a location in a box where they received electrical stimulation to reward-related regions in the brain. They allowed rats to work for this electrical brain stimulation (EBS) by responding on an operant manipulandum (e.g., pressing a lever, spinning a wheel) (Olds and Milner, 1954). The discovery of this technique has been instrumental in mapping reward pathways throughout the brain, and while there are many regions of the brain that can be used to support ICSS (Olds and Milner, 1954; Wise and Bozarth, 1981; Wise, 1996), it is well-documented that stimulation of the medial forebrain bundle (MFB) promotes profound and reliable behavioral outputs (Corbett and Wise, 1980; Pirch et al., 1981; McCown et al., 1986; Tehovnik and Sommer, 1997). Stimulation current parameters can be manipulated to affect the reinforcing value of the EBS and therefore alter ICSS behavior. These parameters include the intensity (i.e., amperes) of the electrical current and the current frequency (i.e., hertz). Elevations in both parameters typically results in increased excitation of the reward-relevant neurons being stimulated, either by increasing the number of neurons engaged by the stimulation (amperes) (Keesey, 1962; Wise et al., 1992) or by increasing the frequency in which a set population of neurons fire (hertz) (Wise and Rompre, 1989; Wise, 2005). Manipulations of current intensity alter the number of neurons activated, i.e., larger current intensities affect a wider population of neurons than smaller currents. Thus, when this parameter is kept constant, the population of neurons excited by EBS is relatively similar regardless of current frequency. The stimulation parameter variable of choice for these protocols is current frequency, as this selection allows us to manipulate the firing rate of the same group of neurons with minimal effects on the time or space of stimulation integration. By manipulating these EBS parameters, we have developed sophisticated models of cost/benefit decision-making that employ ICSS (Rokosik and Napier, 2011, 2012; Tedford et al., 2012; Persons et al., 2013). This application represents a radical departure from the traditionally used reinforcing stimulus (i.e., food) in tasks assessing decision-making in rodents. ICSS may provide several experimental advantages over traditional reinforcement methods. To facilitate operant responding for food, daily intake is often restricted (Feja and Koch, 2014; Hosking et al., 2014; Mejia-Toiber et al., 2014). This practice can confound outcome measures, as there is substantial overlap in the neurobiological systems that are altered during chronic food restriction and those that mediate impulsive decision-making (Schuck-Paim et al., 2004; Minamimoto et al., 2009). Additionally, animals reinforced with food become increasingly satiated throughout a session, which decreases the value of food reinforcement (Bizo et al., 1998), although this effect may be dependent on reinforcer size (Roll et al., 1995). In contrast to food reinforcement, the reinforcer value of the EBS remains stable throughout a session, allowing for more extensive and consistent behavioral assessments (Trowill et al., 1969). This feature allows for testing sessions to occur repeatedly throughout a day, which can be beneficial when studying the effects of pharmacological therapies, specifically chronic drug treatment. Our published probability discounting studies (discussed below) were conducted several times a day throughout chronic dopamine agonist (pramipexole) treatments. We propose that this procedural benefit is more applicable to the human condition and thus provides enhanced translational findings. To date, similar studies assessing dopamine agonist effects on impulsive decision-making using food reward have only assessed acute drug treatments (St Onge and Floresco, 2009; Zeeb et al., 2009; Madden et al., 2010; Johnson et al., 2011; Koffarnus et al., 2011) and it will be of significant interest to compare the behavioral outcomes following both acute and chronic drug treatment between these different reinforcers. While ICSS provides several advantages over food reinforcement, ICSS also presents several disadvantages. For example, ICSS requires invasive brain surgery and recovery, and ill-fitted head stages can result in loss of subjects throughout the behavioral paradigm. Despite these drawbacks, we hold that ICSS is a viable alternative to food reinforcement and presents considerable advantages to food reinforcement in these behavioral tasks.

**Figure 1:**
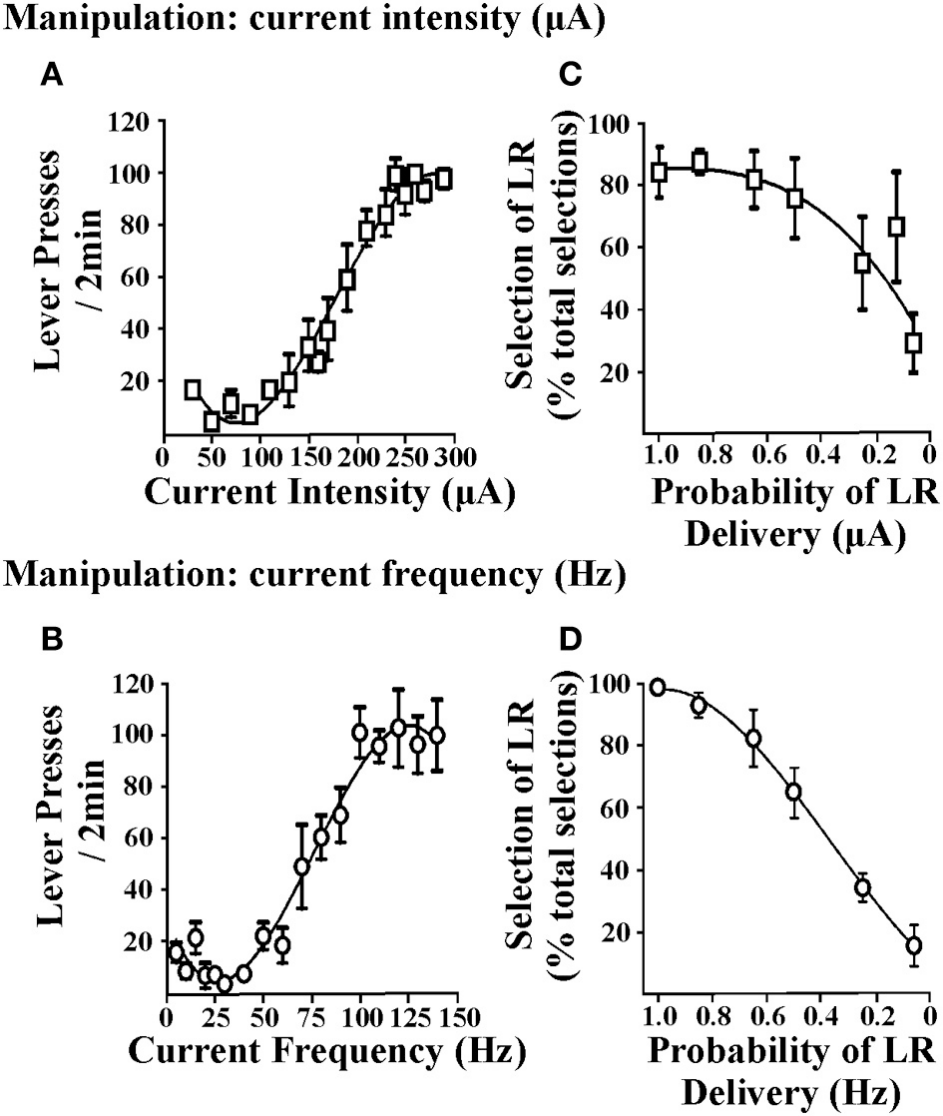
**Effects of brain stimulation parameters on lever-press responding and probability discounting**. The two EBS parameters tested were current intensity and current frequency. Rats lever pressed for EBS (in a fixed ratio-1 schedule of reinforcement) wherein every 2 min, one parameter of EBS was manipulated and the other parameter was held constant. **(A)** Manipulation of current intensity. Current intensities ranging from 10 to 350 μA were presented in randomized order (*n* = 6); current frequency was held at 100 Hz. **(B)** Manipulation of current frequency. Current frequencies ranging from 5 to 140 Hz were presented in randomized order (*n* = 3); current intensity was held constant at a level that was individualized and determined in prior training sessions. Manipulating current intensity or current frequency produced similar patterns of lever-press responding. Data are shown as mean ± s.e.m. for the last three consecutive sessions. Rats were subsequently trained in the probability discounting task and values for the small and large reinforcers were determined individually for each animal by computing the effective stimulation current intensities and current frequencies obtained from the EBS vs. lever-press responding curve that elicited 60 and 90% of maximal lever-press response rates, respectively. Varying the magnitude of current intensity **(C)** or current frequency **(D)** resulted in discounting the large reinforcer (LR) as the probability of delivery was decreased (i.e., decrease in percent selection of the lever associated with the LR over total selections). Data are shown as mean ± s.e.m. for day one of discounting using current intensity and 2 days of discounting using current frequency. Figure modified from Rokosik and Napier (2011) and reprinted with permission from the publisher.

Cost/benefit decision-making tasks require choices to be made between options associated with varying reward magnitudes. Accordingly, reinforcers used in these tasks should demonstrate the ability to produce such changes in reward magnitude and subsequently rats must be able to discriminate between the small reinforcer (SR) and large reinforcer (LR) option. In procedures that use food reinforcement, this is achieved by altering the number of food pellets obtained after a response. In ICSS, the EBS can be varied by changing stimulation current intensity or current frequency. Figure 1 illustrates lever-press responding obtained when current intensity is varied (i.e., current frequency was held constant; Figure 1A) or when current frequency is varied (i.e., current intensity was held constant; Figure 1B). When either parameter is altered, rats exhibit moderate lever pressing for small EBS values and show increased lever-pressing rates for large EBS values, suggesting that the reinforcer value of the larger stimulation is greater (independent of whether current intensity or frequency is manipulated). EBS can therefore be tailored for the small and large reinforcer necessary for cost/benefit decision-making protocols. These reinforcer values can be determined in individual rats by generating stable lever-pressing rate response curves for each animal (Rokosik and Napier, 2011, 2012). Alternatively, a population curve can be generated from a group of rats from which a standardized SR and LR value can be determined (Tedford et al., 2012; Persons et al., 2013). This latter approach provides a more time-efficient and yet reliable means to derive the SR and LR. In a second series of studies, we used either manipulations of current intensity or frequency to establish SR/LR values in a probability discounting task (i.e., risk/reward decision-making). Changes in current intensity reinforcer values (i.e., current frequency was held constant) and current frequency values (i.e., current intensity was held constant) both produce significant discounting behavior in rats (Figures 1C,D). Based in part on the steepness of the discounting curve, current frequency was determined to be the appropriate parameter for manipulating reinforcement values. Once it is established that rats can distinguish between the standardized current frequencies used for the SR and LR, they can be tested in any one of our ICSS-mediated decision-making paradigms: (i) risk/reward decision-making (Rokosik and Napier, 2011, 2012), (ii) delay-based decision-making (Tedford et al., 2012), or (iii) effort-based decision-making (Persons et al., 2013).

## Validating the use of ICSS to evaluate measures of impulsivity and decision-making

The development of new animal models requires careful consideration regarding validity. Thus, in designing these ICSS-mediated decision-making tasks, we have strived to verify face and construct validity, and to ascertain the likelihood for predictive validity.

Face validity refers to the extent in which a test subjectively appears to measure its intended phenomenon. The design of each ICSS-mediated decision-making task was based on current protocols employed in humans for delay and probability discounting (Rasmussen et al., 2010; Leroi et al., 2013) and other effort-based decision-making tasks (Treadway et al., 2009; Buckholtz et al., 2010; Wardle et al., 2011). In humans, measures of cost/benefit decision-making are derived from asking individuals to select between several options available with specific contingencies placed on each selection (i.e., risk, delay, or effort). We emulate this scenario by presenting rats with two simultaneously extended levers, wherein a selection of either lever is associated with small or larger rewards that are also delivered under particular parameters of contingency. Thus, each of our ICSS-mediated decision-making tasks demonstrates face validity.

**Figure 2:**
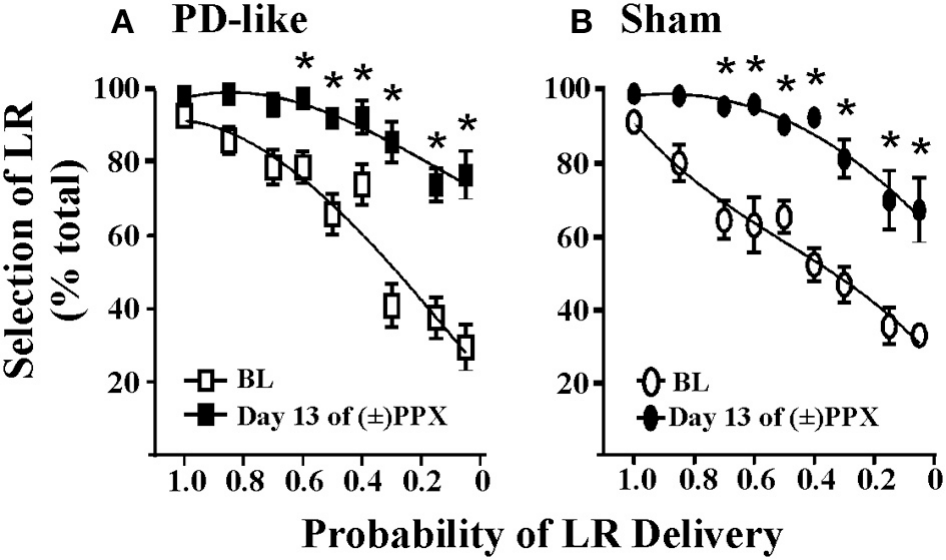
**Effects of pramipexole on risk/reward decision-making using a probability discounting task**. Chronic (±)PPX decreases discounting in PD-like **(A)** and sham control **(B)** rats. Briefly describing the task, PD-like (*n* = 11) and sham control (*n* = 10) rats were trained in the probability discounting task using ICSS. Probabilities associated with delivery of the large reinforcer (LR) were presented in a pseudo-randomized order. Once stable behavior was observed, rats were treated chronically with twice daily injections of 2 mg/kg (±)PPX for 13 days. Data shown were collected from the time point in which we observed the peak effect on the final day of treatment (i.e., 6 h post injection) and are compared with the pretreatment baseline (BL). Shown is the percent selection of the LR (i.e., free-choice ratio) vs. the probability that the LR was delivered. A Two-Way rmANOVA with *post hoc* Newman-Keuls revealed significant increases in % selection of the uncertain, LR following chronic PPX treatment (^*^p < 0.05) for both PD-like and sham rat groups. Although the group averages indicate a PPX-induced increase in suboptimal risk/reward decision-making, two rats in each group showed less than a 20% increase from baseline at the lowest probability tested; therefore, some rats appeared to be insensitive to the ability of the drug to modify probability discounting. Figure modified from Rokosik and Napier (2012) and reprinted with permission from the publisher.

Construct validity refers to the ability of the paradigm to accurately assess what it proposes to measure. In risk/reward and delay-based decision-making, preference for the large reward is decreased as the probability of delivery is lowered, or the delay toward reward delivery is increased, respectively. In effort-based decision-making, individuals demonstrate initial preference for the high effort/large reward option when the effort associated with the large reward is deemed reasonable. A shift in preference to the low effort/small reward is observed when the high effort is no longer worth the energy expenditure. It is well-documented that rodents exhibit similar patterns of risk/reward, delay-based and effort-based decision making compared to humans (Rachlin et al., 1991; Buelow and Suhr, 2009; Jimura et al., 2009), and we have observed these profiles in each of our tasks (Rokosik and Napier, 2011, 2012; Tedford et al., 2012; Persons et al., 2013) (for example, see Figure 2).

Predictive validity refers to the ability of models to foresee future relationships, and we pose that our models can be used to predict the capacity of novel pharmacological treatments to alter cost/benefit decision-making. That is, by demonstrating proof-of-concept through replicating the effects of pharmacological agents on decision-making behaviors that have already been established in humans, we propose that our models may be efficacious in predicting how other drugs may mediate these behaviors in the clinic. For example, a subset of patients with Parkinson's disease (PD) who are treated with dopamine agonist therapies demonstrate an increased prevalence of gambling behavior (Weintraub et al., 2010) and increased discounting in delay-based decision-making (Housden et al., 2010; Milenkova et al., 2011; Voon et al., 2011; Leroi et al., 2013; Szamosi et al., 2013). Thus, our laboratory set out to model PD in rats and study the effects of pramipexole, a commonly employed dopamine agonist associated with gambling behaviors (Weintraub et al., 2010), on cost/benefit decision-making in the rat using the probability discounting task (risk/reward decision-making) (Rokosik and Napier, 2012). To do so, rats were rendered “PD-like” by selective lesioning of dopaminergic terminals within the dorsolateral striatum *via* bilateral infusions of 6-OHDA, while control rats received infusions of the 6-OHDA vehicle (Rokosik and Napier, 2012). Neurons in the dorsolateral striatum of only the 6-OHDA treated rats show a decrease in tyrosine hydroxylase (Rokosik and Napier, 2012), a marker of dopamine. PD-like rats exhibit motor disturbances similar to humans with early-stage PD, which can be reversed dose-dependently with pramipexole treatment. The dose of pramipexole we administered to study risk/reward decision-making alleviates motor deficits, and thus is therapeutically-relevant (Rokosik and Napier, 2012). While we find no difference in baseline “risky” behavior between control rats and PD-like rats, chronic pramipexole treatment increases selection of the risky LR in both groups of rats when probabilities of delivery were small (Figures 2A,B), indicating that pramipexole induces suboptimal risk/reward decision-making. These data concur with studies that have assessed the effects of pramipexole in humans (Spengos et al., 2006; Pizzagalli et al., 2008; Riba et al., 2008). Nonetheless, we infer the predictive validity of our rodent models in indicating other pharmacological agents that may mediate cost/benefit decision-making in humans.

We also have tested mirtazapine, an atypical anti-depressant, in the effort-based decision-making task. Behavioral addictions and substance abuse share many overlapping characteristics, including suboptimal decision-making, and new studies in humans and non-human animals illustrate that mirtazapine is effective at reducing behaviors motivated by abused drugs (e.g., opiates and psychostimulants) even those that are associated with relapse during periods of abstinence (for review, see Graves et al., 2012). Data collected from our ICSS-mediated effort-based decision-making task indicates that mirtazapine effectively reduced preference for a high effort/LR, switching to a low effort/SR, suggesting that the amount of effort required for the LR was no longer “worth it,” or that the reward value of the LR was diminished (Persons et al., 2013). These results suggest that it may be of interest to study the effects of mirtazapine on suboptimal decision-making in problem gamblers in the clinic.

## Conclusion

In summary, we have utilized ICSS as a positive reinforcer in several novel tasks designed to measure separate, yet overlapping, aspects of cost/benefit decision-making exhibited in problem gambling. These measures can be used to further explore the contribution of various neuroanatomical substrates and neurotransmitter systems in problem gambling. ICSS-mediated tasks provide a viable alternative to food reinforcement in these complex operant paradigms. We believe that the validity of these tasks indicates that they can aid in screening drugs for their potential to induce impulse control disorders, such as problem gambling, and to help identify drugs that reduce these disorders.

### Conflict of interest statement

Dr.Napier has received research support from the National Institutes of Health, the Michael J. Fox Foundation and the National Center for Responsible Gaming. Dr. Napier has received compensation for the following: consulting for a not-for-profit health education center and for law offices on issues related to addictions and impulse control disorders; speaking on addictions at community town hall meetings, public high schools, community-based not-for-profits, and professional meetings of drug courts; providing grant reviews for the National Institutes of Health and other agencies; and academic lectures and grand rounds. Dr. Napier is a member of the Illinois Alliance on Problem Gambling, and she provides expert advice on medication development to the Cures Within Research Foundation. Dr. Holtz, Dr. Persons, and Ms. Tedford declare that the research was conducted in the absence of any commercial or financial relationships that could be construed as a potential conflict of interest.
